# 508. A Multidisciplinary Approach to Aftercare Engagement

**DOI:** 10.1093/jbcr/irag033.167

**Published:** 2026-04-06

**Authors:** Kerry Mikolaj, Trudy J Boulter, Brad Jackson, Nichole Schiffer

**Affiliations:** Children's Hospital Colorado, Aurora, CO; Children's Hospital Colorado, Denver, CO; Children's Hospital Colorado, Aurora, CO; Children's Hospital Colorado, Denver, CO

## Abstract

**Introduction:**

Supporting young burn survivors from the time of their injury through their recovery and reintegration into their community is no small feat. This effort takes a dedicated, skilled multidisciplinary team. Pediatric burns are complex due to a child’s growth and development and require ongoing reassessment. The key to aftercare engagement relies on strong therapeutic relationships.

**Methods:**

Our multidisciplinary team includes a provider, nursing, PT, OT, psychology, child life specialist and social work. Once an acute burn is healed, a child returns every 3 months for scar management with our therapy and psych/soc team. During these visits this team builds trust and rapport with the patient and family while outcome measures and therapeutic goals are tracked and assessed. This knowledge guides the therapist’s recommendations for resources the family would benefit from such as school reintegration, community events, burn camps programs, survivor peer support and leadership opportunities. Once aftercare needs are identified, therapy and psych/soc team support patients and families throughout the aftercare continuum.

**Results:**

Through the approach outlined, our center observed an increase in engagement with aftercare services. Patients who regularly attended appointments demonstrated higher participation rates in aftercare programs. The therapeutic alliance built over time between families and the team contributed significantly to families’ willingness to pursue additional resources. Outreach data showed that families who received personalized recommendations from therapists, were more likely to enroll in burn camps and aftercare opportunities. Key indicators of success included increased attendance in aftercare programs year after year, positive family feedback, and improved psychosocial outcomes (PedsQL and Camper Growth Index/Burn Camp Scale) for participating children and families.

**Conclusions:**

The integration of aftercare program referrals into routine follow-up visits is an effective model for engaging pediatric burn survivors in long-term recovery and support. The trust established during ongoing therapy sessions provides a strong foundation for identifying families who may benefit from aftercare services. A coordinated effort by the multidisciplinary team ensures continuity and follow-through, which are critical for successful engagement. This model highlights the importance of proactive, personalized aftercare planning that considers not only physical recovery but also emotional and social reintegration. Aftercare participation, facilitated through this approach, serves as a meaningful step in a child’s holistic healing journey.

**Applicability of Research to Practice:**

Future directions include expanding aftercare capacity and systematically tracking long-term outcomes for aftercare participants and long-term engagement and retention of the multidisciplinary burn team.

**Funding for the study:**

N/A.

